# Gaps in Effective HIV Pre-exposure Prophylaxis Screening and Uptake Among Fishermen in Kenya

**DOI:** 10.1007/s10461-025-04950-1

**Published:** 2025-11-18

**Authors:** Lila A. Sheira, Benard Ayieko, Sarah A. Gutin, Zachary A. Kwena, Edwin D. Charlebois, Kawango Agot, James Moody, Phoebe Olugo, Daniel Adede, Jayne Lewis-Kulzer, Monica Gandhi, Harsha Thirumurthy, Elizabeth A. Bukusi, Carol S. Camlin

**Affiliations:** 1 School of Nursing, Institute for Health and Aging, University of California, San Francisco, 490 Illinois Street, San Francisco, USA; 2 Institute for Global Health Sciences, University of California, San Francisco, USA; 3 Impact Research & Development Organization (IRDO), Kisumu, Kenya; 4 Department of Community Health Systems, School of Nursing, UCSF, San Francisco, USA; 5 Division of Prevention Science, Department of Medicine, University of California, San Francisco, USA; 6 Kenya Medical Research Institute (KEMRI), Kisumu, Kenya; 7 Department of Sociology, Duke University, Durham, USA; 8 Departments of Medicine and of Obstetrics, Gynecology & Reproductive Sciences, School of Medicine, University of California, San Francisco (UCSF), San Francisco, USA; 9 Division of HIV, Infectious Diseases, and Global Medicine, Department of Medicine, University of California, San Francisco, San Francisco, USA; 10 Department of Medical Ethics and Health Policy, Perelman School of Medicine, University of Pennsylvania, Philadelphia, USA

**Keywords:** Men, PrEP, Sexual behavior, Kenya

## Abstract

Despite oral daily HIV pre-exposure prophylaxis (PrEP) effectiveness, uptake remains a challenge. Awareness of one’s HIV status and engagement in prevention is essential to interrupting transmission among populations at high risk of HIV. We explored gaps in HIV risk perception, screening, and PrEP uptake among high HIV-risk fishermen living and working along Lake Victoria, Kenya. Data are from the *Owete* study (NCT04772469), which evaluated the impact of secondary distribution of HIV self-tests and supported linkage to health facilities within social-networks on prevention engagement. Using self-reported sexual behavior data and clinical records, we (1) identified PrEP-eligible men based on self-reported sexual behavior, per Kenya national guidelines; (2) reported the proportion of fishermen who (a) linked to a healthcare facility post-testing, (b) initiated PrEP per clinic records; and 3) analyzed the relationship between PrEP eligibility and perceived HIV risk using logistic regression, controlling for socio-demographic characteristics. Among 733 fishermen, 715 were sexually active in the past 6 months. The median age was 37.2, 68.4% had ≤ primary education, and 87% were married. PrEP eligibility was prevalent (474; 65%); of eligible men, 222 (47%) linked to a healthcare facility after testing, of whom 127 (57%) received a confirmatory HIV test, 31 (14%) initiated PrEP and 2 (0.1%) were already on PrEP. Primary reasons for non-initiation include screening as low risk at the facility (*n* = 66; 30%), declining PrEP (*n* = 41; 18%), and stock-outs (*n* = 5; 2%). Participants reporting moderate or high self-perceived HIV acquisition risk had 1.80 (95% CI: 1.09, 2.98, *p* = 0.023) and 1.75 (95% CI: 1.01, 3.03, *p* = 0.045) times the risk of screening as PrEP eligible, respectively, compared to those reporting no risk. A substantial proportion of fishermen were eligible for PrEP based on sexual behaviors self-reported during the study surveys which align with standardized PrEP eligibility criteria. Nevertheless, we identified a high discordance between these self-reported criteria which renders one PrEP eligible and actually screening PrEP-eligible at a healthcare facility, highlighting potential gaps in effective screening for and subsequent coverage of PrEP to men at risk for HIV acquisition. Understanding the potential limitations of current PrEP screening protocols, as well as clients’ experiences with PrEP screening in the clinic, may increase the sensitivity of PrEP screening and close gaps in effective PrEP coverage.

*Clinical Trial Registration*: NCT04772469

**Supplementary Information:**

The online version contains supplementary material available at 10.1007/s10461-025-04950-1.

## Introduction

HIV pre-exposure prophylaxis (PrEP) has the potential to substantially decrease HIV incidence globally if used effectively and among populations at greatest risk of HIV acquisition [1]. The most ubiquitous PrEP method globally is a daily oral pill combining tenofovir disoproxil fumarate (TDF, 300 mg) and emtricitabine (200 mg). While highly effective at preventing HIV acquisition, oral PrEP’s effectiveness is diminished with suboptimal adherence, and thereby ranges from 30 to 99% [2]. Suboptimal PrEP adherence is well-documented among many key populations for HIV prevention in Eastern and Southern Africa, including male and female sex workers [3, 4] and men who have sex with men (MSM) [5]. Known structural barriers, including anticipated stigma related to disclosure of sexual activities to providers [6, 7] and inability to seek care during offered clinic hours [8, 9] undermine PrEP continuation. Oral daily PrEP’s potential coverage is further diminished due to low awareness and subsequent uptake [7], despite the World Health Organization having recommended oral PrEP as part of a comprehensive HIV prevention package in 2015, and its subsequent global rollout [10]. While there are newer PrEP technologies which may support adherence, including long acting injectables such as cabotegravir and lenacapavir, these technologies are either not widely available or still undergoing regulatory review and approvals. Moreover, even after these newer regimens become available, oral daily PrEP will remain a vital prevention tool due to the need for coverage when long acting injectables are discontinued, not preferred by clients, or are cost-prohibitive at a large scale.

Cascades or continuums in HIV treatment and care have been widely implemented to identify progression among standardized benchmarks of HIV care provision and treatment outcomes, identify gaps in implementation, and inform policy [11–13]. These frameworks have informed the development of parallel HIV prevention and pre-exposure prophylaxis cascades of care, whereby benchmarks include missed opportunities in PrEP provision among PrEP-eligible individuals and not being retained in PrEP care [14, 15]. The cascade can also be expanded to include two benchmarks preceding screening and initiation of PrEP: awareness and willingness [16]. These cascades, in turn, have been used to identify gaps in PrEP care among key populations [15]. For example, in Kenya, the location of our study, a 2020 study reported missed opportunities for PrEP screening, ranging from 45% among MSM to 78% among adolescent girls and young women [17]. Another study in Kenya among MSM found poor sensitivity of a eligibility screening per national guidelines (6.6% screening eligible), yet high (69.7%) uptake of PrEP [5]. There are less data on eligibility, linkage, and uptake among other key populations in Kenya, such as fisherfolk [18]. 

While there is significant research highlighting barriers to continuation of and adherence to PrEP among PrEP initiates across numerous key populations for HIV prevention [4, 7, 19, 20], research identifying gaps in PrEP initiation, the first benchmark, is more challenging to quantify given potential selection bias in those who may seek out PrEP at public health facilities compared to those who may never seek PrEP out, as well as low sensitivity of the PrEP screening tools [21]. In most settings for HIV preventative care provision, receiving PrEP follows a multi-step, standardized process including screening within a health facility via a nationalized behavioral risk assessment, an HIV test to confirm HIV seronegative status, and laboratory assessments to evaluate kidney function and to evaluate for concomitant conditions of Hepatitis C. Often, these procedures happen in the same area as routine care provided to people living with HIV (PLHIV), may involve long wait times, and the packaging of PrEP once dispensed is physically similar to that of antiretroviral therapy (ART). These characteristics exacerbate stigma associated with seeking HIV care or being assumed to be a PLHIV. Modifications to this process have taken place in more recent years where PrEP is offered during antenatal care visits for prevention of vertical transmission of HIV [22]. Taken together, screening and dispensation characteristics are commonly cited as barriers impeding engagement in PrEP care.

Among populations with an elevated risk of HIV acquisition in East Africa, a high HIV-burdened area, fishermen working alongside Lake Victoria are less likely to engage in HIV preventative and treatment care and to be aware of their HIV status [8, 23]. The estimated prevalence of HIV among fishermen in this area is 29% [24]. Low engagement in care is partially attributed to characteristics of their work, whereby fishermen work irregular hours at beach locations far from public health clinics [23, 24]. This is coupled with lower engagement in HIV care among the general population of men, who are less likely to test for HIV, engage in HIV treatment and PrEP, and experience poor HIV treatment outcomes [25–27]. 

This study sought to understand gaps in PrEP screening and linkage among fishermen working and living in a high HIV burdened region of Kenya enrolled in a larger cluster randomized controlled trial (cRCT) of a secondary HIV self-test kit (HIVST) distribution intervention.

## Methods

### Study Design

We leverage data from *Owete*, a cRCT (NCT04772469) evaluating the impact of an HIV status-neutral, social network-based intervention including the distribution of HIV self-tests kits on HIV testing, linkage to HIV treatment ART or prevention (oral PrEP) among fishermen working along the shores of Lake Victoria, Kenya. The study protocol has been described in detail elsewhere; [28] in brief, we conducted a census and subsequent mapping of social networks among fishermen working in three beach communities along Lake Victoria in Siaya County, Kenya. Fishermen were eligible if they were (1) age ≥ 18 years; (2) listed in the Beach Management Unit registry, a requirement for engaging in the occupation of fishing; and (3) not participating in another HIV-related study. After the mapping and identification of social networks (clusters) and within cluster leaders, a baseline survey was conducted among all fishermen mapped to be part of a social network.

Social networks (clusters and their network central “promoter”) were randomized 1:1 to the intervention or control condition. Promoters were invited to a full day training on HIV knowledge, and benefits of HIV testing, prevention and treatment; promoters in the intervention arm were invited to a second day of training on use of HIVST kits. The intervention arm promoters were given HIVSTs to distribute to each member of their social network; the study did not track the actual distribution of the HIVST kits by the promoter, but rather if they were received among the cluster members. The kits were affixed with information for a study-affiliated clinic for linkage, as well as information regarding a monetary voucher for travel (500 Kenyan Shillings or ~$4 USD in 2022) available upon linkage to the clinic, independent of their test result or HIV status. Promoters in the control arm received vouchers with information about routine HIV testing at nearby clinics to distribute to men in their networks. Promoters in both arms were trained in using an HIV status-neutral approach to encourage men in their networks to test for HIV and link to prevention (PrEP) if HIV-negative or treatment (ART) if found to test positive for HIV.

The primary parent study outcomes were self-reported HIV testing via (1) any modality and (2) via HIVST at three months and are reported elsewhere [29]. Other data collected include HIVST and voucher distribution, as well as linkage to public health facilities, and were measured at three months post training via surveys and health facility records at partnered health facilities. Surveys were interviewer-administered and included questions about demographic characteristics, mental health, wealth, HIV knowledge and risk perception, and food security. As part of the baseline and three-month surveys, participants also completed a “Relationship History Calendar” (RHC), whereby sexual behavior (e.g. number of partners, condom use, transactional sex) was collected per partner month. This tool has been found to reduce social desirability bias in the reporting of sexual behaviors [30, 31]. 

### Participant Identification

We sought to identify study participants in terms of the following phases of the PrEP care continuum: (1) PrEP-eligible men based on self-reported sexual behavior per Kenya national guidelines; (2) of these, the proportion of fishermen who a) linked to a healthcare facility post-testing; (3) of these, the proportion of men who initiated PrEP per clinic records, and (4) of these, the proportion who had tenofovir urine levels consistent with adherence to PrEP [32]. Finally, a secondary aim of the sub-study was to analyze the relationship between PrEP eligibility (Step 1 of the continuum) and perceived HIV risk.

### Variables

At the time of analysis the Kenyan National AIDS and STI Control Programme (NASCOP) guidelines for an HIV Risk Assessment for PrEP initiation included the following criteria [33]: (a) sex with more than one person; (b) sex without a condom; (c) sex with someone of an unknown HIV status; (d) if partners are at risk for HIV; (e) sex with someone who is at risk of HIV; (f) history of sexually transmitted infection(s); (g) if they desire pregnancy; (h) post-exposure prophylaxis use; (i) injecting drug-user; and/or (j) engaging in transactional sex. The full HIV screening assessment is included in Supplementary Table 1. An affirmative answer to any of the individual criteria results in an individual being categorized as eligible. Our study collected data on items a, b, e, and j, which are among the most frequent methods of HIV acquisition among fishermen [24]. For item a, we assessed overlapping sexual partnerships within the month for any of the prior six months. For item b, we included those men who reported any condomless sex and >1 sexual partner. For items d and j, participants were able to identify partners as casual, one-night stands, sex workers, and/or *jaboya/jakambi* (a form of transactional sex involving fish for sex trade) [34] and are combined given overlapping domains of high-risk and transactional sex. Linkage and PrEP initiation were dichotomous variables developed from administrative data received from the study-affiliated clinics on participants’ linkage status.

PrEP adherence was measured via a novel, point of care urine assay which measures urine tenofovir levels. This assay is a noninvasive way to measure PrEP adherence consistent with PrEP usage over the past 4–7 days, and will detect tenofovir in the urine of 90% of adherent individuals over this time period [32, 35]. The variable is a dichotomous variable quantifying the presence of tenofovir in the urine levels vs. no detection; urine was collected among those who initiated PrEP or self-reported PrEP use at 3-months post baseline. For perceived HIV risk, we asked participants “*what do you think your chances are of acquiring HIV in the future*”; response options were none, low, moderate, high, or not applicable, as well as don’t know and decline to respond. The variable is analyzed as a categorical variable.

### Analysis

Summary statistics of the entire sample and those who screen eligible for PrEP at study baseline are reported. We then report proportions at each phase of the PrEP care and treatment continuum as described. The study was status neutral at baseline; i.e., men living with HIV could receive HIVST kits and could link to return unused test kits or redeem their voucher; HIV seropositivity was later reported at follow up either via facility records among those fishermen living with HIV who linked or those who self-reported their status at follow-up. HIV status was not questioned in the baseline survey. We therefore report two sets of data: (1) data independent of HIV status (given status neutrality) and (2) data excluding individuals whom we later determined to be living with HIV.

Two generalized linear models with the Poisson family and the log link were estimated to model risk ratios between self-perceived HIV risk and being PrEP eligible; this model controlled for the Beach (one of three sites along Lake Victoria in Siaya county from where participants were recruited), age (per year), educational attainment (primary or less [reference], some or complete secondary, and post-secondary or higher), marital status (married [reference], in a relationship but not married, or divorced/widowed/single) and intervention arm (intervention vs. control); robust standard errors were included to account for the clustering of participants within social networks. One model was run with the entire sample assuming neutral status; the second model excludes those whom we determined were living with HIV.

## Results

Among the 733 men enrolled at study baseline, the median age was 37.2 years (interquartile range 19.7, 82.3). Most men were married (85.4%), and 22.0% of married participants were in polygynous relationships; 68.4% had attained a primary level or less education, and 84.9% reported fishing as their primary source of income (Table 1).

**Table 1:** Socio-demographic characteristics of Owete study participants and sub-study participants at baseline, assuming HIV status neutrality (*n* = 733)

Characteristic	Not PrEP eligible (*n* = 260)	PrEP eligible (*n* = 474)	Total (*n* = 733)	*p*-value
Age, years (median, IQR)	35.4 (29.0, 42.2)	35.8 (30.2, 42.5)	37.2 (19.7, 82.3)	0.52
Marital Status				< 0.0001
Married	200 (76.9%)	426 (90.1%)	626 (85.4)	
Unmarried but partnered	15 (5.8%)	15 (3.2%)	30 (4.1)	
Single/divorced/widowed	45 (17.3%)	32 (6.8%)	77 (10.5)	
Education				0.068
Primary or less	168 (64.9%)	332 (70.3%)	500 (68.4)	
Some/all secondary	70 (27.0%)	120 (25.4%)	190 (26.0)	
>Secondary	21 (8.1%)	20 (4.2%)	41 (5.6)	
Polygynous marriage^2^	15 (7.5%)	123 (28.9%)	138 (22.0)	< 0.0001
Fishing is primary source of income	215 (82.7%)	407 (86.0%)	622 (84.9)	0.38
Food insecure	244 (93.8%)	434 (91.8%)	678 (92.5)	0.30
Health Status				0.94
Very good	36 (14.0%)	73 (15.5%)	109 (15.0)	
Good	113 (43.8%)	204 (43.3%)	317 (43.5)	
Fair	96 (37.2%)	173 (36.7%)	269 (36.9)	
Poor	13 (5.0%)	21 (4.5%)	34 (4.7)	
Sexual risk behavior past 6 months				
Any concurrent sexual partner^4^	0 (0%)	279 (59.9%)	279 (40.2)	< 0.0001
Any high risk sexual partner^5^	8 (3.3%)	78 (16.5%)	86 (12.0)	< 0.0001
No condom use with any partner	228 (94.2%)	453 (95.8%)	680 (95.2)	0.35
Ever tested for HIV	249 (95.8%)	463 (97.9%)	712 (97.1)	0.12
Every heard of HIV self-testing	199 (76.5%)	347 (73.4%)	546 (74.5)	0.51
Perceived risk of HIV acquisition				< 0.0001
None	24 (9.3%)	18 (3.81%)	42 (5.74%)	
Low	142 (54.8%)	212 (44.8%)	354 (48.4%)	
Moderate	24 (9.3%)	95 (20.1%)	119 (16.3%)	
High	12 (4.6%)	45 (9.5%)	57 (7.8%)	
N/a	47 (18.2%)	86 (18.2%)	133 (18.2%)	
Don’t know	10 (3.9%)	17 (3.6%)	27 (3.6%)	

^1^Using self-reported data on sexual relationships as a proxy

^2^Asked of married men

^3^Defined as an AUDIT-C score of 4 or greater

^4^Two or more overlapping sexual partners within any given month over the past six months

^5^Sexual partner reported as casual, commercial sex worker, one night stand, inherited partner, or stranger

Nearly all (715, 97.5%) reported any sexual partnerships within the previous six months and thus completed the baseline relationship history calendar. Overall, 473 participants (64.5%) had self-reported behaviors consistent with screening as PrEP eligible, including having concurrent sexual partners (279, 40.2%), condomless sex (680, 95.2%), and/or sex with someone at risk of HIV or engaging in transactional sex (92, 12.9%). Almost all men reported having ever tested for HIV over their lifetime (712, 97.1%), with 71.3% reporting their last test within the prior year.

### PrEP Cascade of Care

**Fig. 1:**
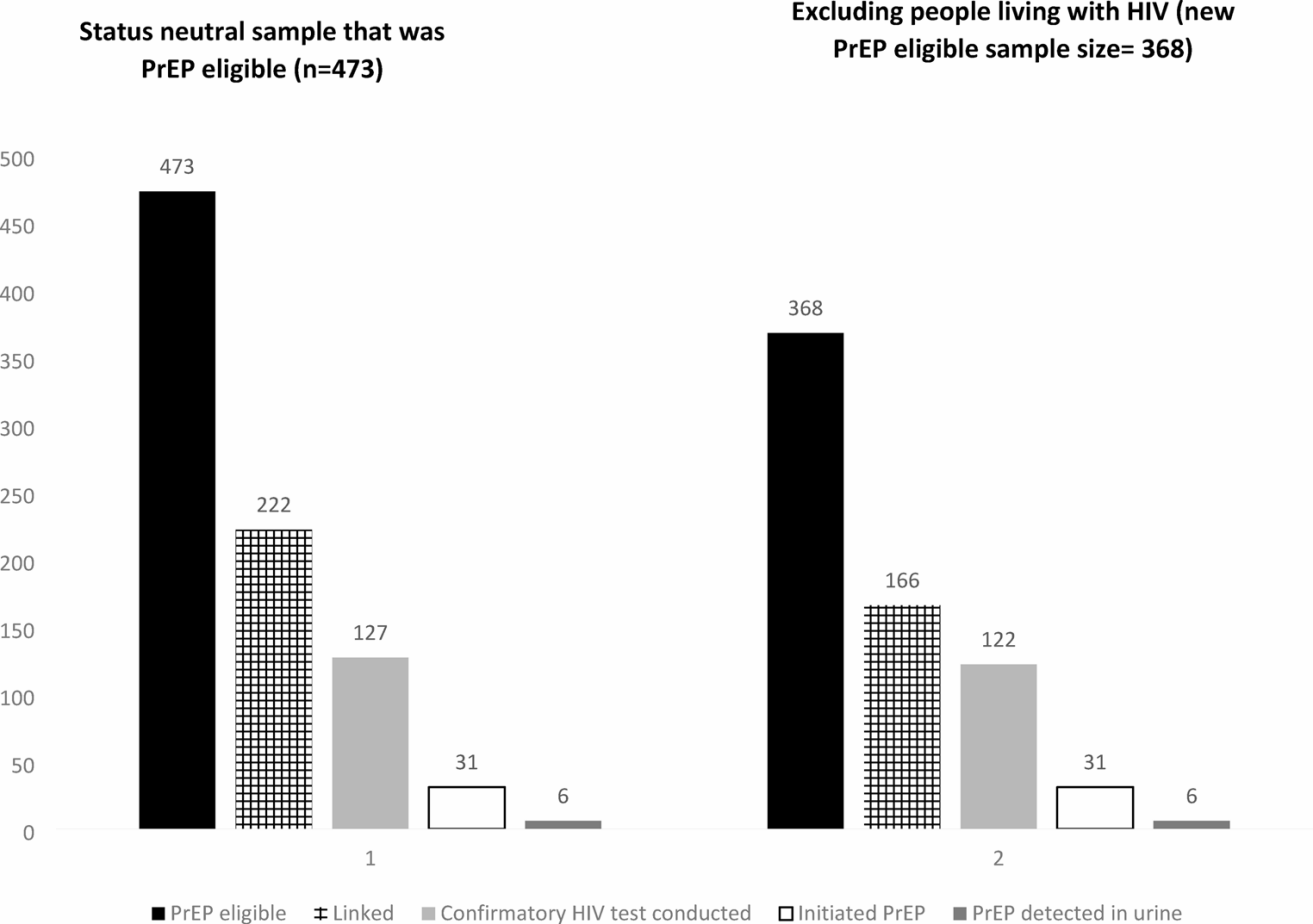
Cascade of PrEP care, Owete study

Among men whom we identified as having behaviors consistent with being PrEP eligible (*n* = 473), 222 (46.9%) linked to a healthcare facility following HIV testing (compared to an overall 384 or 52.4% of total overall sample linked), of whom 127 (57.2%) conducted a confirmatory HIV test. While study protocol dictated that all HIV seronegative men be screened for PrEP, only 31 (14.0%) of those who linked initiated PrEP (Fig. 1). There were a total of 44 (of 473, 9.3%) PrEP users in the study sample. Of these, 31 were participants who initiated PrEP during their linking to the health facility, of whom we conducted analyses of urine tenofovir levels, and 13 were PrEP users who were ascertained during medical chart abstraction. Of the 31 PrEP initiators, 6 (19.4%) had urine samples consistent with tenofovir exposure. Among those who did not initiate PrEP, leading reasons included being screened as not at risk (*n* = 66, 29.8%), declining PrEP (*n* = 41, 18.5%), and PrEP being out of stock (*n* = 5, 2.3%).

### Excluding Individuals Living with HIV

The intervention was HIV status neutral at baseline, meaning HIV status was not ascertained during any baseline assessment. However, during the study, we were able to determine that 167 (22.8%) were living with HIV, of whom only 1 was a seroconversion occurring within the previous three months. Of these 167 participants living with HIV, 105 (22%) were previously categorized as being “PrEP eligible” based on self-reported sexual behavior. In the scenario excluding these participants from the estimate of individuals eligible for PrEP, the number of PrEP eligible participants is reduced to 368 participants or 64.2% (out of an updated denominator of 573). Of the individuals who were not known to be living with HIV, 166 (45.1% of all PrEP eligible) linked to a healthcare facility, of whom 122 (73.5% of those who linked) conducted a confirmatory HIV test, and 31 (18.7%) initiated PrEP. Among those who did not initiate PrEP, leading reasons included being screened as not at risk (*n* = 64, 38.6%), declining PrEP (*n* = 38, 22.9%), and stock outs (*n* = 5, 3.0%).

### Perceived HIV Risk

Participants whom we categorized as PrEP eligible were twice as likely to report a high (9.5% vs. 4.6%) or moderate (20.1% vs. 9.3%) self-perceived risk of HIV compared to those who we did not categorize as PrEP eligible. In unadjusted models, participants with moderate and high HIV risk had 1.86 (95% confidence interval [CI] 1.32, 2.62, *p* < 0.0001) and 1.84 (95% CI: 1.31, 2.59, *p* < 0.001) times the risk of screening as PrEP eligible. In adjusted models, the association was slightly attenuated to 1.80 (95% CI: 1.09, 2.98, *p* = 0.023) and 1.75 (95% CI: 1.01, 3.03, *p* = 0.045) times the risk of screening as PrEP eligible among those whose self-perceived HIV risk was moderate and high, respectively, compared to those who reported no self-perceived risk of HIV acquisition. When excluding those living with HIV, the magnitude of the association increased to 1.90 (95% CI: 1.11, 3.25, *p* = 0.019) and 1.82 (95% CI: 1.02, 3.30, *p* = 0.044) times the risk of screening as PrEP eligible among those whose self-perceived HIV risk was moderate and high, respectively, compared to those who reported no self-perceived risk of HIV acquisition (Table 2).

**Table 2:** Unadjusted and adjusted associations between self-perceived HIV acquisition risk and PrEP eligibility (*n* = 733)

	Status = neutral model		Excluding known PLHIV	
	Unadjusted model^1^	Adjusted model^1,2^	Unadjusted model^1^	Adjusted model^1,2^
Self-perceived HIV risk				
None	Ref.	Ref.	Ref.	Ref.
Low	1.40 (0.99–1.96)	1.35 (0.83–2.18)	1.48* (1.02–2.15)	1.42 (0.85–2.37)
Moderate	1.86** (1.32–2.62)	1.80* (1.09–2.98)	2.00** (1.38–2.89)	1.90* (1.11–3.25)
High	1.84** (1.31–2.59)	1.75* (1.01–3.03)	1.93** (1.34–2.78)	1.83* (1.02–3.30)
Not applicable	1.51* (1.07–2.12)	1.41 (0.85–2.36)	1.92** (1.28–2.88)	1.80 (0.93–3.48)
Don’t know	1.47 (1.00–2.17)	1.43 (0.74–2.78)	1.67* (1.10–2.53)	1.56 (0.78–3.12)

*** <0.0001, **p < 0.01, *p < 0.05

^1^Modified Poisson models were modelled to estimate the risk ratio

^2^Models adjusted for participant age, educational attainment, marital status, and fishing beach where recruited. Models also have robust standard errors to account for clustering at the level of the social network

## Discussion

PrEP has the potential to significantly curb incident HIV cases if offered and used effectively and particularly among populations which face heightened vulnerabilities to HIV acquisition. Our study found that among a population of fishermen working alongside Lake Victoria, Kenya, 65% of participants were potentially PrEP eligible, yet 18% of those potentially eligible—or only 4% of the total study population—initiated PrEP, with substantial declines in engagement across the PrEP cascade of care. Importantly, we also found that a substantial proportion of men found from our community-based survey data to be at risk on the basis of their reported sexual behaviors, thus fitting criteria for eligibility for PrEP, were screened as ineligible for PrEP once in a health facility (38.6%). These findings corroborate prior evidence demonstrating potential self-selection and social-desirability bias in seeking sexual health care in public health settings [36, 37], while also raising questions about the process (and its sensitivity) for determining PrEP eligibility.

Across high HIV-burdened countries of Eastern and Southern Africa, eligibility assessment for PrEP consists of provider-administered assessments of risk, usually in the form of a behavioral assessment or checklist of risk consisting of questions around sexual behaviors, injecting drug use, and/or the status of sexual partners. This process is accompanied by HIV testing to ascertain HIV seronegative status, as well as blood tests to assess kidney and liver function, prior to PrEP dispensation. Given our study was status neutral at baseline, we had the opportunity to identify participants living with HIV who screened eligible for PrEP. While HIV seropositivity precludes PrEP eligibility, we interpret these findings as the individual being a potential beneficiary of sero-discordant partner programming. This finding highlights a potential gap in HIV care, whereby HIV preventative care may be separated from those already living with HIV. In the era of Undetectable = Untransmittable, this work highlights how PrEP screening may be integrated into standard of HIV care, especially among individuals who are virally non-suppressed, to prevent onward transmission of HIV. The sensitivity of the PrEP screening process, i.e., the ability of the screener to identify an individual who is truly PrEP eligible, is generally unknown, with substantial potential repercussions if low (i.e., individuals truly eligible for PrEP are misclassified as not eligible). If we were to consider our self-reported questionnaires as a “true positive”, the screeners conducted among our participants had a sensitivity between 19% (those who initiated) and 73% (those who conducted confirmatory tests), as we could not ascertain individuals whom the clinic screened as PrEP eligible but who declined to initiate. Moreover, we observed a potential false negative rate of the clinic-based behavioral assessment of 30–39%, whereby individuals self-reported behaviors consistent with being PrEP eligible (via a community-based survey) but were recorded as not being at risk (via provider-administered screening checklist) in the health care facility.

Our data demonstrate that both individual and health facility factors may lead to missed opportunities for PrEP dispensation among individuals at risk of HIV acquisition, who may in turn become preventable incident HIV cases. For example, while low perceived risk of HIV acquisition is generally acknowledged as an underestimation of true risk [38, 39], among our study participants, moderate and high self-perceived risk of HIV acquisition was associated with being PrEP eligible. This self-perception of moderate and high HIV acquisition risk did not, in turn, translate to uptake of PrEP. The second largest gap in the PrEP care cascade that we observed occurred between confirmatory HIV testing and PrEP uptake, most likely during the behavioral screener. Identifying individuals at risk of HIV acquisition focuses on individual behaviors, however a more comprehensive screener that improves self-report of sexual behaviors, accounts for relationship factors (i.e., suspected or known sexual concurrency of one’s partner(s), engagement in *jaboya* [34] specifically which one may not identify as transactional sex), and incorporates the community burden of HIV (e.g., lessens PrEP eligibility criteria in high HIV burdened communities) may be more sensitive statistically in identifying those who could benefit from PrEP.

The largest gap in the PrEP care cascade occurred between uptake and adherence, consistent with numerous studies documenting suboptimal PrEP adherence levels and refills among PrEP initiates, undermining PrEP’s potential prophylactic effects [2, 5, 40]. Numerous well-documented barriers explain suboptimal adherence to PrEP, including anticipated stigma or being perceived as living with HIV, transportation barriers, and/or fear of being seen with PrEP pills [20, 41]. Objective measures of PrEP adherence such as those used in this study are thus critical for measurement and for providing direct adherence support to PrEP users. Indeed, within the *Owete* study we documented discordance between self-reported and objective measures of PrEP adherence [42]. Newer PrEP modalities, such as long-acting injectable PrEP, may address many of these known barriers to adherence. Nevertheless, HIV screening protocols with poor sensitivity may undermine the potential impact of these newer modalities if they are not offered to those who may benefit most from PrEP.

Our study’s findings must be interpreted within their limitations. Our study team was not privy to the PrEP screening process of the participants and thus cannot determine why individuals we determined to be PrEP-eligible were not screened as such. We know that social desirability bias, especially within healthcare settings, results in underreporting of sexual risk behaviors [43]. It is plausible that our participants, having already built rapport with our study team and/or by conducting the surveys in their communities rather than within a health facility, felt more comfortable disclosing sexual behaviors in the study. Another limitation of our data collection system is that we did not comprehensively collect data on all of the PrEP screening criteria, including questions around injecting drug use and HIV sero-discordant relationships, as well as awareness, the first benchmark on the expanded PrEP cascade of care. This underreporting would result in an underestimation of the PrEP eligible sample. These limitations are balanced by our study’s strengths, including being among the first studies to conduct an analysis of the PrEP cascade of care [44], and among fishermen, a population facing dual vulnerabilities in HIV risk acquisition due to the mobile nature of their work and the fact that men are less likely to know their HIV status compared to women [45, 46]. 

## Conclusions

We identified a high discordance between self-reported sexual behavior consistent with being PrEP eligible and screening as PrEP-eligible at a clinic, highlighting potential gaps in effective screening for and subsequent coverage of PrEP among a population of fishermen working alongside Lake Victoria, Kenya. With the advent of newer PrEP technologies in the approval pipeline, improving the sensitivity of PrEP behavioral screening procedures can ensure that PrEP screening and prescription can support effective PrEP coverage, in turn reducing preventable HIV cases.

## Supplementary Information

Below is the link to the electronic supplementary material.

Supplementary material 1 (DOCX 14.5 kb)

## Data Availability

The data that support the findings of this study are available on request from the corresponding author. The data are not publicly available due to privacy or ethical restrictions.

## Abbreviations

ART: Anti-retroviral therapy
cRCT: Cluster randomized controlled trial
HIV: Human immunodeficiency virus
NASCoP: National AIDS and STI Control Programmen
PrEP: Pre-exposure prophylaxis
TDF: Tenofovir disoproxil fumarate
