# Supplementary material for: Gaps in Effective HIV Pre-exposure Prophylaxis Screening and Uptake Among Fishermen in Kenya
Source: AIDS Behav. 2025 Nov 18;30(4):1211–20. doi: 10.1007/s10461-025-04950-1 (PMC12875317; doi:10.1007/s10461-025-04950-1)
Supplement: Supplementary file 1 — Supplementary material 1 (DOCX 14.5 kb) [file 10461_2025_4950_MOESM1_ESM.docx]

| **Supplemental Table 1: HIV Screening questions**  *Screening question refer to the past 6 months & include:* |
| --- |
| 1. **“Have you had sex with more than one person?” *** 2. **“Have you had sex without a condom?” *** 3. “Have you had sex with anyone whose HIV status you do not know?” 4. **“Are any of your partners at risk of HIV?” *** 5. “Have you had sex with a person who has HIV?” 6. “Have you received a new diagnosis of a sexually transmitted infection?” 7. “Do you desire pregnancy?” ** 8. “Have you used or wanted to use PEP or PrEP for sexual exposure to HIV?” 9. “Have you injected drugs that were not prescribed by healthcare provider? If yes, did you use syringes, needles or other drug preparation equipment that had already been used by another person?” 10. **“Have you received money, housing, food or gifts in exchange for sex?” *** 11. “Have you been forced to have sex against your will?” 12. “Have you been physically assaulted, including assault by a sexual partner?” *** |

*Indicates which questions were used for this analysis.

**Indicates which questions were N/A.

***Indicates questions that were not applicable to this study.
